# Blood oxidative stress biomarkers in women: influence of oral contraception, exercise, and N-acetylcysteine

**DOI:** 10.1007/s00421-022-04964-w

**Published:** 2022-06-08

**Authors:** Karlee M. Quinn, Llion Roberts, Amanda J. Cox, David N. Borg, Evan N. Pennell, Daniel R. McKeating, Joshua J. Fisher, Anthony V. Perkins, Clare Minahan

**Affiliations:** 1 Griffith Sports Science, Griffith University, Gold Coast, QLD 4222 Australia; 2 Sport Performance Innovation and Knowledge Excellence Unit, Queensland Academy of Sport, Nathan, QLD 4111 Australia; 3 School of Medical Science, Griffith University, Gold Coast, QLD 4222 Australia; 4 Menzies Health Institute Queensland, Griffith University, Gold Coast, QLD 4222 Australia; 5 School of Human Movement and Nutrition Sciences, University of Queensland, Brisbane, QLD 4072 Australia; 6 The Hopkins Centre, Menzies Health Institute Queensland, Griffith University, Brisbane, QLD 4102 Australia

**Keywords:** Antioxidants, Female athlete, Inflammation, Performance, Physiology, Ventilation

## Abstract

**Purpose:**

To compare physiological responses to submaximal cycling and sprint cycling performance in women using oral contraceptives (WomenOC) and naturally cycling women (WomenNC) and to determine whether N-acetylcysteine (NAC) supplementation mediates these responses.

**Methods:**

Twenty recreationally trained women completed five exercise trials (i.e., an incremental cycling test, a familiarisation trial, a baseline performance trial and two double-blind crossover intervention trials). During the intervention trials participants supplemented with NAC or a placebo 1 h before exercise. Cardiopulmonary parameters and blood biochemistry were assessed during 40 min of fixed-intensity cycling at 105% of gas-exchange threshold and after 1-km cycling time-trial.

**Results:**

WomenOC had higher ventilation (*β* [95% CI] = 0.07 L·min^−1^ [0.01, 0.14]), malondialdehydes (*β* = 12.00 mmol·L^−1^ [6.82, 17.17]) and C-reactive protein (1.53 mg·L^−1^ [0.76, 2.30]), whereas glutathione peroxidase was lower (*β* =  22.62 mU·mL^−1^ [− 41.32, − 3.91]) compared to WomenNC during fixed-intensity cycling. Plasma thiols were higher at all timepoints after NAC ingestion compared to placebo, irrespective of group (all *p* < 0.001; *d* = 1.45 to 2.34). For WomenNC but not WomenOC, the exercise-induced increase in malondialdehyde observed in the placebo trial was blunted after NAC ingestion, with lower values at 40 min (*p* = 0.018; *d* = 0.73). NAC did not affect cycling time-trial performance.

**Conclusions:**

Blood biomarkers relating to oxidative stress and inflammation are elevated in WomenOC during exercise. There may be an increased strain on the endogenous antioxidant system during exercise, since NAC supplementation in WomenOC did not dampen the exercise-induced increase in malondialdehyde. Future investigations should explore the impact of elevated oxidative stress on exercise adaptations or recovery from exercise in WomenOC.

**Supplementary Information:**

The online version contains supplementary material available at 10.1007/s00421-022-04964-w.

## Introduction

Prolonged high-intensity exercise is one stimulus that transiently disrupts the pro-oxidant and antioxidant (i.e., redox) environment, often resulting in oxidative stress and damage (Powers and Jackson 2008; Thirupathi and Pinho 2018). While an acute increase in exercise-induced oxidative stress may be important for training adaptations (Gomez-Cabrera et al. 2008), excessive production of reactive oxygen and nitrogen species (RONS) during prolonged exercise has been associated with skeletal muscle fatigue (Barclay and Hansel 1991; Powers and Jackson 2008). Thus, exercise generated RONS can be described by a bell-shaped (hormesis) curve, whereby there are two endpoints of physiological function (Reid et al. 1993). Another factor that may moderate performance is the effect of oral contraceptives (OC) on the redox environment during exercise. Irrespective of training status, OC-use has been consistently associated with oxidative stress and damage measured at rest via blood biomarkers, e.g., elevated malondialdehyde (MDA) and low lipid-soluble antioxidants (Cauci et al. 2016; De Groote et al. 2009; Kowalska and Milnerowicz 2016; Palan et al. 2006; Quinn et al. 2018; Quinn et al. 2021). Therefore, it could be postulated that oxidative stress is exacerbated in women using OC compared to women naturally cycling during high-intensity exercise. This concurrent OC- and exercise-induced oxidative stress may drive a rightwards shift on the hormesis curve and affect skeletal muscle function and subsequent performance. However, there is paucity of experimental research in women, particularly research that controls for female sex hormones when investigating physiological responses to exercise.

Although several studies have found that OC use increases oxidative stress at rest, only one study has examined the exercise-induced change in lipid peroxidation in women using OC (Massart et al. 2012). Massart et al. (2012) found that MDA significantly increased in both OC-users and non-users after a combat (Judo) training session, but the percent magnitude of change (i.e., pre- to post-exercise) was greater in OC non-users. However, the implications that this has on performance was not investigated (Massart et al. 2012), and tightly controlled research that accounts for relative work performed is required to clarify the influence of OC-use on exercise-induced oxidative stress. Characterising these physiological responses to exercise in more detail is important given that approximately 50% of female athletes use OC (Larsen et al. 2020b).

In an effort to understand the effect of RONS on skeletal muscle function, study designs have employed the use of antioxidant supplementation (Matuszczak et al. 2005; Paschalis et al. 2018). Associated findings suggest that when the concentration of exercise-generated RONS is high, the administered antioxidant may protect muscle cells against RONS-related fatiging mechanisms (e.g., calcium sensitivity) and thus enhance performance (Andrade et al. 2001; Matuszczak et al. 2005). One such antioxidant is N-acetylcysteine (NAC), which has been shown to protect cells against oxidative insults by acting as a cysteine donor for the (re)synthesis of glutathione, mitochondrial sulfane sulphur production and/or to a lesser extent, a direct scavenging ability (Aldini et al. 2018). In exercise physiology research conducted predominately in men, NAC has been established as an ergogenic aid in hand-grip exercise (Matuszczak et al. 2005), intermittent high-intensity running (Cobley et al. 2011; Rhodes et al. 2019) and high-intensity cycling exercise (McKenna et al. 2006; Medved et al. 2004b; Paschalis et al. 2018; Slattery et al. 2014). More recently, the use of NAC as an ergogenic aid has been demonstrated to be more effective in male participants with a glutathione deficiency compared to those with normal or high glutathione levels (Margaritelis et al. 2020; Paschalis et al. 2018). As such, the available research suggests that NAC supplementation may have a positive impact on exercise performance for women using OC given there is preliminary evidence demonstrating low glutathione and glutathione peroxidase (GPx) in OC-users at rest (Kowalska and Milnerowicz 2016; Quinn et al. 2021).

Our primary aim was to determine whether there are differences in blood biomarkers of oxidant damage and antioxidants during heavy, fixed-intensity exercise in women naturally cycling compared to women using OC. We were also interested in exploring whether acute (i.e., 1 h prior to exercise) NAC supplementation alters blood biomarkers during fixed-intensity cycling in the two groups, and whether there was any subsequent effect on 1-km cycling time trial (TT) performance. It was hypothesized that oxidant damage would change similarly for both groups in response to exercise; however, women using OC would have higher absolute oxidant damage measured in the blood after exercise compared to naturally cycling women. It was also hypothesized that NAC supplementation would dampen the increase in biomarkers of oxidant damage in both groups, however, improve cycling TT performance only in women using OC.

## Methods

### Participants

**Fig. 1:**
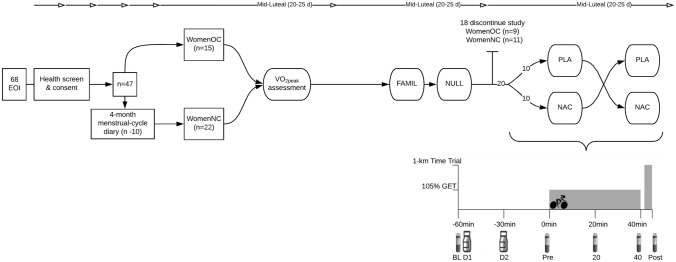
Schematic of study design and protocol. Investigation of plasma redox responses to fixed-intensity cycling at 105% of gas exchange threshold (GET) and an all-out 1-km cycling time-trial in women with natural, regular menstrual cycles (WomenNC) and women using combined, monophasic oral contraceptives (WomenOC) after ingestion of a placebo beverage (PLA) and beverage containing N-acetylcysteine (NAC) supplied in two doses (D1 and D2). Blood samples were acquired at five timepoints (BL, Pre, 20, 40 and Post). All participants were familiarized (FAMIL) to the experimental protocols and performed a replicate of the intervention trials without consuming any beverage (NULL). Menstrual cycle phase was controlled for in this study design (i.e., data collected during confirmed mid-luteal phase); top arrows with open triangle denotes start of new menstrual cycle. EOI; Expressions of Interest

There were 68 expressions of interest received from women aged 18–35 years to participate in the present study (Fig. 1). Women were screened and excluded if they were determined as ‘high-risk’ to vigorous exercise, using an antioxidant or vitamin supplement, smoked, using any form of hormonal contraception other than monophasic, combined OC or had a history of endocrinological, musculoskeletal, inflammatory, respiratory or cardiovascular disorders as determined by all stages of the Adult Pre-Exercise Screening Tool (Exercise and Sports Science Australia, 2019). As such, 47 women were eligible and subsequently stratified into two groups: (1) women with natural menstrual cycles (WomenNC; *n* = 22) and (2) women using combined, monophasic oral contraception for at least 12 months (WomenOC; *n* = 15). WomenNC were asked to complete a 4-month menstrual-cycle diary to determine average menstrual cycle length and regularity. Ten WomenNC were excluded from further participation due to irregular menstrual cycles. Of the remaining 37 screened volunteers, seventeen women ceased their participation part-way through the study for reasons, such as pregnancy, skeletal muscle injury, development of an irregular menstrual cycle after screening or commencing hormonal contraception partway through the study. Therefore, 20 women (WomenNC; *n* = 11 and WomenOC; *n* = 9) that were classified as recreationally active (Decroix et al. 2016) successfully completed all five stages of this experimental investigation. All women provided written informed consent before commencing the study, which was approved by the Griffith University Human Research Ethics Committee, Queensland, Australia.

### Verification of OC and menstrual cycle phase

WomenOC and WomenNC completed all trials during the active pill-taking days (days 13–18 of active tablets) and during the mid-luteal phase of the menstrual cycle, respectively. The primary outcome variables (blood oxidative stress biomarkers) are thought to be altered in OC-users due to metabolism of the exogenous sex hormones, rather than from the resulting decrease in endogenous sex hormones. Therefore, the allocated testing days permitted comparison between the groups when they were exposed to exogenous (ethinyl-estradiol and progestin) or endogenous (estradiol and progesterone) female sex hormones. WomenNC were tested in the mid-luteal phase given this is the only phase that both estradiol and progesterone are elevated simultaneously. WomenOC were tested as late into the active pill-taking days as possible (days 13–18) to minimise data collection during transient shifts in biomarker concentrations that can occur within the first week of pill-taking days after a withdrawal bleed (Quinn et al. 2021) while minimising the risk of symptoms that may influence exercise effort/motivation in the last week of active pill taking days (Sulak et al. 2000).

Average menstrual cycle length was calculated from four consecutive months and used in a predictive equation for determining the approximate time of the luteinizing hormone (LH) surge (Mcintosh et al. 1980). This information was used to determine the appropriate testing days for the preliminary experimental trials. For the intervention trials, where key physiological parameters were examined, this information was accompanied with colorimetric enzyme immunoassay urinary LH testing to predict ovulation (One Step Ovulation Test, Home Health Diagnostics, UK) and a blood sample for *post priori* determination of female sex hormone concentrations. WomenNC performed the urinary LH testing at home according to the manufacturer’s instructions and photographed the test strip so it could be visually inspected by the primary investigator to control for subjective interpretation. A positive test indicated, with a level of confidence of 95%, that ovulation would occur 14–26 h later (Miller and Soules 1996). The mid-luteal phase was defined as 6–11 days after predicted ovulation and the mid-luteal phase was later verified by a progesterone concentration of ≥ 16 nmol·L^−1^ (Janse De Jonge et al. 2019), which was measured in the participant’s first intervention trial.

### Study design

Participants reported to the laboratory on five separate occasions to perform an incremental cycling test to exhaustion, a familiarization session, an experimental trial without any intervention (NULL), and two experimental trials with an intervention (i.e., intervention trials; see Fig. 1 for schematic). Two trials could be performed during one menstrual cycle (i.e., mid-luteal phase testing window of 5 days). Therefore, laboratory testing was conducted over three consecutive months, to enable consistency in female-sex hormone ratios (i.e., high endogenous estrogen and progesterone) in WomenNC, and was replicated for WomenOC given known differences in oxidative stress between weeks of OC use (Quinn et al. 2021). The exercise protocol included a 40-min heavy, fixed-intensity cycling bout immediately followed by an all-out 1-km cycling TT. A two-phasic exercise protocol was implemented to enable the investigation of female-specific physiology in a controlled exercise setting, as well as assess performance related outcomes. The intervention trials included the consumption of a placebo beverage, or a beverage containing N-acetylcysteine (NAC), that were performed as an acute dosing, double-blind randomized crossover study design. Previous intervention trials investigating the effects of NAC on exercise performance have implemented multiple-day oral NAC protocols (Cobley et al. 2011; Paschalis et al. 2018; Rhodes et al. 2019; Slattery et al. 2014) or with intravenous infusion (Matuszczak et al. 2005; McKenna et al. 2006; Medved et al. 2004b). However, these longer antioxidant supplementation and wash-out protocols can be problematic with female-specific experimental research due to the methodological intricacies of controlling for menstrual cycle phase (Janse De Jonge et al. 2019). Furthermore, there can be undesirable dampening of training adaptations associated with chronic antioxidant supplementation regimens (Gomez-Cabrera et al. 2015). Thus, an acute (same-day) oral supplementation protocol was selected as the most appropriate for the present study. The experimental trials were performed at 07:00 (± 2 h) to minimise the influence of circadian rhythm, and laboratory temperature (22–23 °C) and humidity (55–65%) remained constant.

### Dietary and exercise constraints

Participants were instructed to maintain their normal training and dietary practices throughout the study. An antioxidant intake food-frequency questionnaire (Braakhuis et al. 2011) that surveys habitual intake of 70 food items over 1 month was completed on two occasions i.e., during the NULL trial and the first intervention trial. All subjects completed a 24 h food record and 7-day exercise diary before every test, including questions of subjective sleep quality and muscle fatigue. Participants were instructed to replicate these food and exercise regimes before testing, as well as have at least 3 days of rest from muscle-damaging exercise (i.e., eccentric dominant or unaccustomed exercise), and 24 h of complete rest leading into each trial. These questionnaires were monitored by the primary investigator and two tests were postponed for reasons relating to muscle-damaging exercise within the 3-day criteria.

### Incremental cycling test to exhaustion

Participants performed an incremental cycling test to exhaustion on a cycle ergometer (Lode Excalibur Sport, Groningen, Netherlands) to determine $\dot{\mathrm{V}}$ O_2_peak and the gas exchange threshold (GET). The Lode handle bar, seat height and position was individualized for each participant and these dimensions were recorded to be reproduced in subsequent trials. Participants completed a 10-min cycling warm-up at 50 W, after which, advanced into an incremental work rate of 20 W·min^−1^ until the participant reached volitional exhaustion. A self-selected pedal cadence between 70 and 80 rev·min^−1^ was maintained throughout. Beat-by-beat heart rate (Polar Electro, Australia) and breath-by-breath gas exchange (Ultima, CardiO2, PMedical Graphics, St. Paul, MN) were acquired continuously during the test, while rate of perceived exertion (RPE) was recorded on each minute (Borg 1982). $\dot{\mathrm{V}}$ O_2_peak was considered as the highest 30-s rolling average of oxygen consumption achieved during the incremental cycling test. Two experienced researchers independently determined GET using traditional V-slope methods (Beaver et al. 1986), and recorded the corresponding power output for each participant. When there was a discrepancy (*n* = 3), a third researcher evaluated the data and an average of the three values was obtained.

### Familiarisation and NULL trial

A familiarization trial was conducted to minimise learning effects and ensure participants were accustomed to the testing procedures that would be replicated in the NULL, placebo and NAC trials. The NULL trial was completed 3–4 days after the familiarization trial, which served to quantify physiology and performance outcomes without any intervention. Participants cycled on a Lode ergometer for 40 min at a power output corresponding to 105% of GET. This design permitted each individual to complete the same relative amount of total work that was of sufficient intensity (Leaf et al. 1997; Sacheck et al. 2003) and duration (Alessio et al. 1997) to induce redox alterations prior to completing the performance trial. The all-out 1-km cycling TT was completed on a Wattbike cycle ergometer (Wattbike Pro, Nottingham, UK). The magnetic resistance was set at zero, while the air resistance was self-selected with the guidance of an accredited sports scientist and these settings, along with the Wattbike dimensions were replicated for each 1-km cycling TT.

The reliability of 1-km TT performance was determined from seven participants who had data available from both the familiarization and NULL trial. Performance in the 1-km TT was shown to have good reliability, with an intraclass correlation coefficient (2, *k*) of 0.90 [95% confidence interval, CI 0.62–0.98] and a SEM of 1.7 s (Weir 2005). Based on this SEM, the minimum difference for a worthwhile change was calculated as 4.8 s, and this value was considered the smallest effect size of interest (Lakens et al. 2018).

### Intervention trials

Participants arrived at the laboratory after an 8-h overnight fast. A 23G indwelling catheter (Insyte™, Becton–Dickinson, BD, Juiz de Fora, MG, Brazil) was inserted into the participant’s antecubital vein by a trained phlebotomist using standardised procedures. Five venous blood samples were collected per trial for determination of female sex-steroid concentrations (first trial only for luteal phase confirmation) and blood redox biochemistry (both trials). Sampling occurred before consuming any beverage (baseline; BL), 60 min after consuming the first beverage and before commencing exercise (Pre), 20 min into fixed-intensity exercise (EX_20_), 40 min into fixed-intensity exercise (EX_40_) and 2 min after completing the 1-km time trial (Post). Dose one and dose two of the beverage containing placebo or NAC were delivered at 60 min and 30 min prior to exercise, respectively. Heart rate, RPE, blood lactate concentration and exactly 2 min of gas exchange were acquired at Pre (i.e., at rest), EX_20_ and EX_40_. On completion of fixed-intensity cycling, the gas analysis ceased and participants were transferred to the Wattbike within 2 min. The participants were instructed to complete the 1-km TT in the fastest time possible while remaining in the saddle, with no other instruction, with the exception of strong verbal encouragement during the trial. Performance parameters were recorded from the computer monitor attached to the Wattbike. Measurements of peak heart rate (i.e., determined as the highest heart rate recorded during the time trial), peak RPE (obtained immediately post-exercise) and blood lactate concentration (defined as the highest value measured from a capillary earlobe sample at 1 min or 3-min post-exercise) were obtained. Participants returned to the laboratory 3–4 days later to perform identical procedures after consuming the alternate beverage (placebo or NAC).

### Placebo and N-acetylcysteine supplementation

Participants consumed a commercially available beverage (Powerade Ion4 Lemon Lime, The Coca-Cola Company, USA), of which the total volume was calculated relative to body mass so that carbohydrate intake equated to 0.5 g·kg^−1^ per trial (i.e., < 50 kg = 415 mL; 50–54 kg = 455 mL; 55–59 kg = 500 mL; 60–64 kg = 540 mL; > 65 kg = 590 mL). Each beverage was mixed with 6 teaspoons of commercially available lemon juice (Zesty Lemon Juice, Woolworths, Australia; i.e., placebo trial) or with lemon juice and powdered NAC (i.e., NAC trial). Pilot work in a group of individuals that did not partake in the experimental trials (*n* = 10) demonstrated that the taste-matched placebo beverage was indistinguishable from the NAC beverage. NAC was batch tested (HASTA, Melbourne, Australia) after being purchased in a powder formula from Bulk Nutrients (Batch: 4006 2203, Tasmania, Australia) and weighed on electronic scales (AUX Analytical Balance, Shimadzu, Japan) in two separate quantities; dose one: 70 mg·kg^−1^ of body mass of NAC and, dose two: 35 mg·kg^−1^ of body mass of NAC. Previous literature suggests that ingestion of NAC when it is i) solution-based compared to capsule-based and, ii) at least 70 mg·kg^−1^ of body mass 1 h before exercise, is sufficient to elevate cysteine-related thiols in the plasma and result in a performance improvement (Corn and Barstow 2011; Ferreira et al. 2011). The first dose was mixed in 60% of the total beverage volume and dose two in 40% of the total beverage volume to be delivered at 60 min and 30 min prior to exercise, respectively. A two-dose protocol was used to control for between-participant rate of beverage consumption as well as increase the likelihood of NAC to be elevated in the plasma during the 1-km cycling TT (Corn and Barstow 2011). The beverage was delivered in a double-blinded fashion (one researcher not involved in the day-to-day administration of trials was not blinded for safety precautions). All participants were naive to NAC supplementation before commencement of the study, and participants were monitored for any adverse symptoms.

### Preparation and analysis of venous blood samples

Approximately 15 mL venous blood was collected at each timepoint from the indwelling venous cannula and distributed into EDTA and serum separation tubes (Becton–Dickinson, BD, Juiz de Fora, MG, Brazil). All tubes were gently mixed by inversion. Immediately after collection at each timepoint, the serum separation tube was left to clot at room temperature for 30 min before centrifugation for 10 min at 1500 × g and 4˚C, whereas the EDTA tube was immediately centrifuged using the same protocol. Plasma and serum were stored in aliquots and frozen at -80 ˚C for subsequent analysis.

All biochemistry parameters were analysed in duplicate, with the exception of total antioxidant capacity (TAC), which was analysed in triplicate. Serum oestradiol (Cat. no 33540, Beckman Coulter, Australia) and progesterone (Cat. no 33550, Beckman Coulter, Australia) were assessed using an automated clinical immunoassay analyser (Access 2, Beckman Coulter, Australia) as per the manufacturer’s instructions. The intra-assay coefficient of variation (CV) was 4.04%. Serum liver and metabolite biochemistry were assessed using an automated clinical chemistry analyser (AU480, Beckman Coulter, Australia) according to the manufacturer’s instructions. The serum biochemistry intra-assay CV was 2.6%. Plasma GPx (colorimetric; ab102530), free thiols (fluorometric; ab112158), MDA (ab118970), 3-nitrotyrosine (3NT; ab210603; Abcam Australia Pty Ltd, Melbourne, Australia) and total glutathione (ADI-900–160; Sapphire Bioscience Pty.Ltd, Redfern, Australia) were determined using commercially available kits as per manufacturer’s instructions. The intra-assay CV was 2.6%, 6.4%, 8.0%, 4.8% and 4.6%, respectively.

### Total antioxidant capacity assay

As described previously (Re et al. 1999), 2,2'-azino-bis(3-ethylbenzothiazoline-6-sulfonic acid (ABTS) was dissolved in water to a concentration of 7 mmol·L^−1^. ABTS radical cation (ABTS^•+^) was then produced by adding 2.45 mmol·L^−1^ potassium persulfate. The mixture was left in the dark at room temperature for 12–16 h before use. ABTS^•+^ stock solution was diluted to an absorbance of 0.7 at 750 nm using either phosphate buffered saline (PBS; pH 7.4) for the study of hydrophilic antioxidant activity or ethanol for the study of lipophilic antioxidant activity. Plasma samples (2 µL of straight plasma; 10 µL of lipophilic extract) were added to a 96-well flat bottom plate in triplicate. Trolox (6-hydroxy-2,5,7,8-tetramethychroman-2-carboxylic acid) was used as a standard and dissolved in PBS or ethanol (final concentration 0–40 µM). Diluted ABTS^•+^ solution (190 µL) was added to all wells and absorbance measurements were taken at 750 nm at 30 °C, 5 min after the initial mixing. Measurements were made using a Tecan Sunrise Absorbance Reader with Magellan Standard software (TECAN, Austria). Decolourisation of the assay is linear with increasing concentrations of the standard, Trolox. The results were expressed relative to Trolox activity (mmol·L^−1^ Trolox equivalent). The intra-assay CV was 3.9%.

### Data analyses

All analyses were performed using R (version 4.0.3) (Team 2013). Participant characteristics were compared using independent *t* tests. Heart rate, expired gas variables (i.e., oxygen consumption, ventilation, respiratory exchange ratio), gross efficiency (calculated according to Brouwer 1957; Moseley and Jeukendrup 2001) and lactate during fixed-intensity cycling were analysed using linear mixed-effect models (Kuznetsova et al. 2017). Candidate models included all two-way interactions between *Time* (or *Time*^2^), *Group (levels: WomenNC and WomenOC)* and *Supplement (levels: Placebo and NAC)*, or the three-way interaction between these variables; and participant as a random effect variable—intercept, or intercept and slope (supplement). Cadence was included as a standardised covariate (mean 0, SD 1) for expired gas variable models. Final models were selected based on the lowest Bayesian Information Criteria (BIC) value. RPE data were fit using beta-regression, in a Bayesian framework (Liu and Kong 2015). A normal (mean 0, precision 0.001) prior distribution was set for the regression coefficients and a Uniform (mean 0, SD 20) prior for the SD of the random effect.

A series of candidate linear mixed-effect models were fit to each biochemical variable (i.e., GPx, thiols, TAC, total glutathione, MDA, 3NT, CRP, albumin), with the final model chosen based on the lowest BIC value. The candidate models ranged from: a base model, which included *Time* (or *Time*^2^), *Group*, and *Supplement* as fixed effects (i.e., no interaction terms), to a model with all possible interactions, between *Time* (or *Time*^2^), *Group* and *Supplement*. All models included a random intercept and slope (supplement) for each participant.

The unscaled difference in 1-km TT completion time between placebo and NAC (*y*_*i*_) was calculated for each participant (*y*_*i*_ = NAC_*i*_ – placebo_*i*_) and modelled with *Group* as a fixed factor. Equivalence tests were used to determine whether the within, and between, group difference in completion time fell inside the SESOI (Lakens et al. 2018). When the 90% CI of the parameter estimate for group (i.e., *β*_Group_) was within the bounds of 0 ± 4.8 s, it was concluded that 1-km performance was statistically equivalent (Lakens et al. 2018).

When appropriate, pairwise contrasts were made, with *p* values adjusted (Tukey) (Lenth et al. 2020). Cohen’s *d* was calculated for the standardized difference between groups, supplements or timepoints ‘*k*’ and ‘*l*’ (Cohen 1992). The pooled SD was used as the denominator of *d*. Data are reported as the mean and 95% CI, unless otherwise stated. The α for all tests was set at 5%.

## Results

### Participant characteristics

Serum oestradiol and progesterone concentrations were statistically lower in WomenOC than WomenNC (Table 1). All other participant characteristics were not statistically different between groups, including $\dot{\mathrm{V}}$ O_2_peak (Table 1). WomenNC had an average cycle length of 30 ± 3 days, and all women were confirmed to have ovulated ~ 6–11 days before commencing the intervention trials (Janse De Jonge et al. 2019). There were three types of progestin’s consumed by WomenOC; Drospirenone (*n* = 3), Cyproterone acetate (*n* = 3) and Levonorgestrel (*n* = 3). The concentration of progestin ranged from 150 to 300 µg and the concentration of ethinyl–oestradiol ranged from 30 to 35 µg.

**Table 1:** Participant characteristics of women with natural, regular menstrual cycles (WomenNC) and women using combined, monophasic contraceptives (WomenOC)

Variable	WomenNC (*n* = 11)	WomenOC (*n* = 9)	*p* value
Age (year)	25 ± 4	24 ± 3	0.804
Body mass (kg)	64.1 ± 9.2	67.8 ± 8.7	0.365
Height (m)	1.69 ± 0.08	1.7 ± 0.06	0.408
Body mass index (kg·m^2^)	22.41 ± 2.48	22.80 ± 2.13	0.736
Blood pressure (mmHg)			
Systolic	108 ± 12	106 ± 12	0.667
Diastolic	66 ± 10	64 ± 8	0.689
Total cholesterol (mmol·L^−1^)	3.87 ± 0.95	4.01 ± 0.65	0.777
Fasting glucose (mmol·L^−1^)	3.33 ± 0.89	3.31 ± 0.39	0.954
HDL (mmol·L^−1^)	1.53 ± 0.23	1.50 ± 0.33	0.978
Oestradiol (pmol·L)	543.76 ± 202.02	63.54 ± 30.17	< 0.001
Progesterone (nmol·L)	27.59 ± 13.51	3.37 ± 1.58	< 0.001
Dietary antioxidant intake (mmol·d^−1^ across 2-mo)	10.66 ± 6.97	10.06 ± 4.69	0.934
Training (h·week^−1^ ≥ moderate-intensity)	7 ± 2	7 ± 2	0.954
$\dot{\mathrm{V}}$ O_2_peak (mL·kg^−1^·min^−1^)	42.34 ± 5.81	39.70 ± 3.71	0.220
$\dot{\mathrm{V}}$ O_2_peak (L·min^−1^)	2.71 ± 0.52	2.70 ± 0.20	0.781
Peak heart rate (beats.min^−1^)	187.1 ± 5.1	183.4 ± 9.1	0.273

Values ± SD

*HDL* high-density lipoprotein, $\dot{\mathrm{V}}$*O*_*2*_*peak* peak oxygen uptake

### Adverse events

No major adverse reactions were reported by any participant. However, two participants reported mild gastrointestinal discomfort after 2 h of NAC ingestion and another participant after 5 h of NAC ingestion. All symptoms had resolved within the same day.

### Fixed-intensity cycling physiology and RPE

**Fig. 2:**
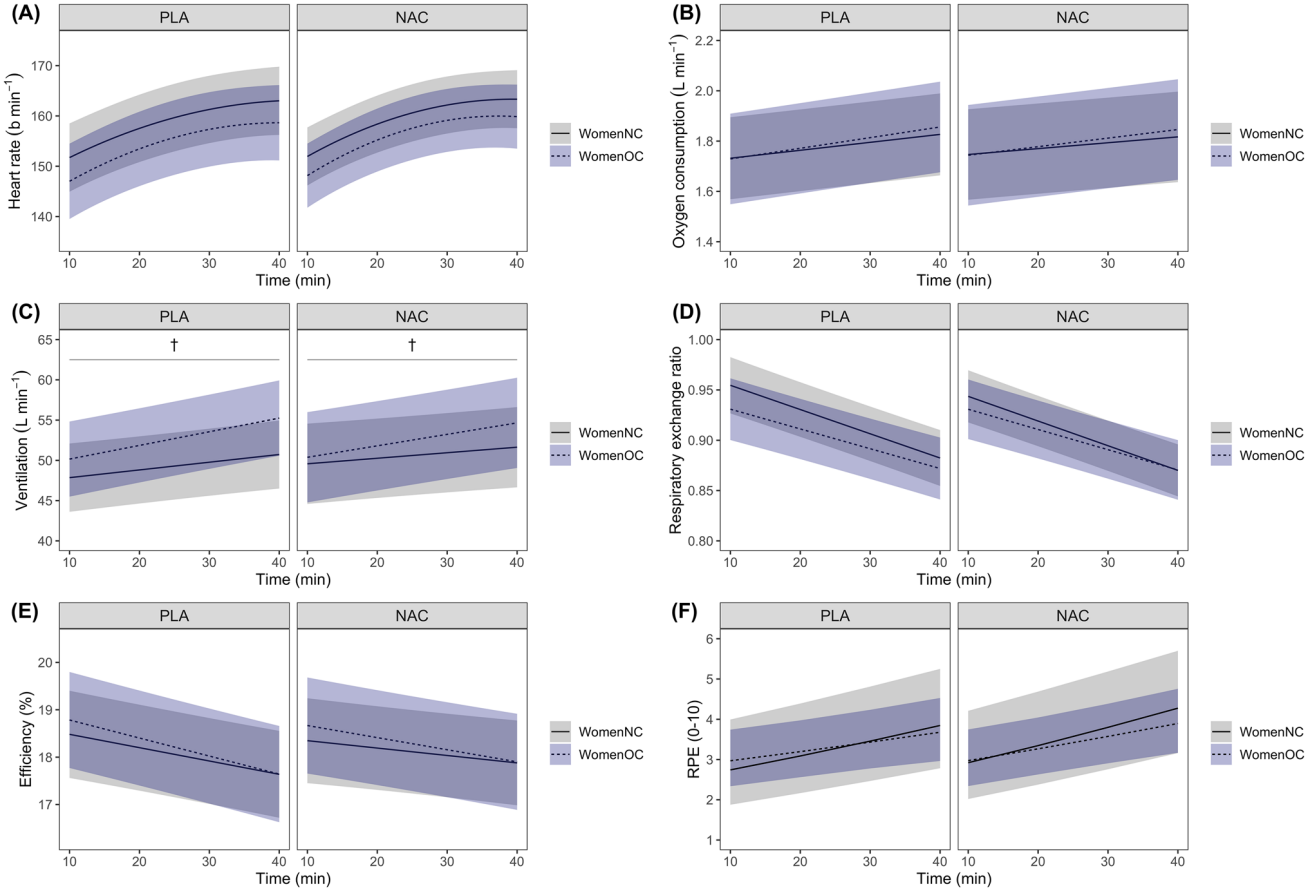
Heart rate (**A), **expired gas variables (**B**–**D**), efficiency (**E**), and rating of perceived exertion (RPE; **F**) response during 40 min of fixed-intensity cycling in with natural, regular menstrual cycles (WomenNC) and women using combined, monophasic oral contraceptives (WomenOC), after ingestion of a placebo beverage (PLA) and a beverage containing N-acetylcysteine (NAC). Solid (WomenNC) and dashed (WomenOC) lines indicate the mean response and shaded ribbons show the 95% confidence interval. Heart rate, oxygen consumption, and RPE increased with time, while respiratory exchange ratio decreased; however, these variables were not statistically different between groups or supplements. Ventilation (C) increased with time, and to a greater extent in WomenOC compared to WomenNC (*β* = 0.07 L·min^−1^ [0.01, 0.14]), irrespective of supplementation (indicated by a dagger; †). Efficiency (E) decreased with time; but possibly to a lesser extent in the NAC compared to placebo trial, irrespective of group (*β* = 0.012% [− 0.001, 0.026])

Heart rate increased with *Time* (Fig. 2A), but was not different between *Supplement* or *Group*. Oxygen consumption increased with time (*β* = 0.003 L·min^−1^ [0.002, 0.004]), but was not different between *Group* or *Supplement* (Fig. 2B). Ventilation increased during cycling (*β* = 0.10 L·min^−1^ [0.04, 0.15]), and the magnitude of increase was greater in WomenOC compared to WomenNC (*β* = 0.07 L·min^−1^ [0.01, 0.14]), irrespective of supplementation (Fig. 2C). Respiratory exchange ratio decreased over time (*β* = − 0.0024 [− 0.0029, − 0.0019]), but was not different between *Group* or *Supplement* (Fig. 2D). There was an effect of *Time* (*β* = − 0.03% [− 0.04, − 0.02]) on efficiency (Fig. 2E). There may also have been a *Time* by *Supplement* effect on efficiency (*β* = 0.012% [− 0.001, 0.026]), with efficiency possibly preserved over time in the NAC compared to placebo trial, irrespective of group (Fig. 2E).

RPE increased during cycling (logit *β* = 0.017 [0.011, 0.023]), but was not different between *Group* or *Supplement* (Fig. 2F). Lactate increased as a result of exercise, but was not different between Ex_20_ and Ex_40_, or between *Group* or *Supplement* at any timepoint. For WomenNC, mean [95% CI] lactate at pre-exercise, Ex_20_ and Ex_40_ during placebo was 1.5 [0.8, 2.1], 3.3 [2.6, 3.9] and 3.1 [2.4, 3.8], respectively; and during NAC was 1.4 [0.7, 2.1], 3.2 [2.5, 3.8] and 3.0 [2.3, 3.7], respectively. For WomenOC, lactate at pre-exercise, Ex_20_ and Ex_40_ during placebo was 1.6 [0.8, 2.3], 3.2 [2.5, 3.9] and 3.1 [2.3, 3.8], respectively; and during NAC was 1.6 [0.9, 2.3], 3.2 [2.4, 3.9] and 3.0 [2.3, 3.8], respectively.

### Biochemical variables

**Fig. 3:**
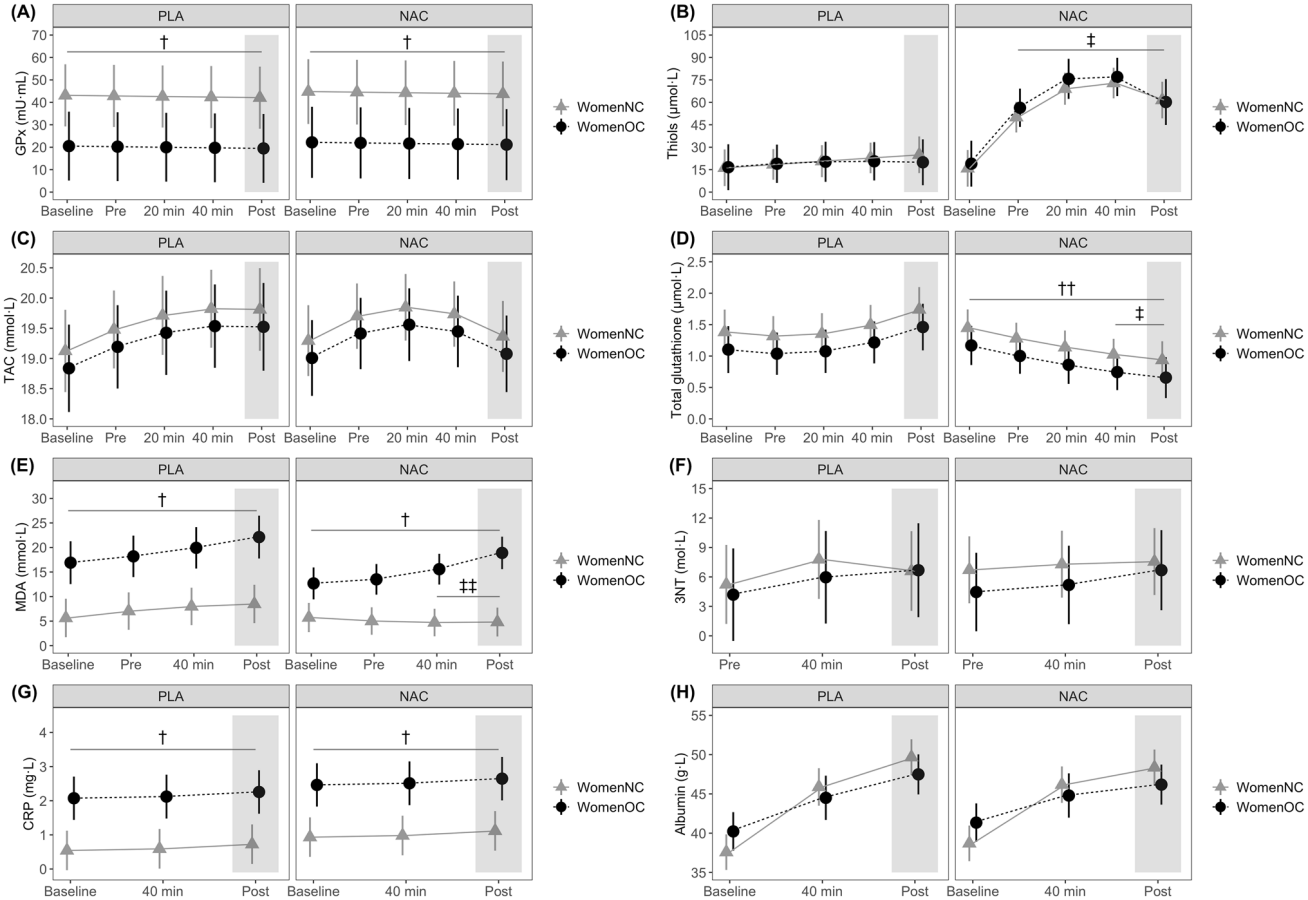
Time course changes of biochemical variables in naturally cycling women (WomenNC) and women using combined, monophasic oral contraceptives (WomenOC) from baseline to pre-exercise (Pre) i.e., 1 h after ingestion of a beverage containing N-acetylcysteine (NAC) or placebo (PLA), during (20 min) or at termination of a submaximal, fixed-intensity 40-min cycling task (40 min), and after a 1-km cycling time-trial (Post; grey shaded area). Biochemical variables: glutathione peroxidase (GPx; **A**), plasma thiols (**B**), total antioxidant capacity (TAC; **C**), total glutathione (**D**), malondialdehyde (MDA; **E**), 3-nitrotyrosine (3NT; F), C-reactive protein (CRP; **G**) and albumin (**H**). † statistically different between WomenNC and WomenOC, i.e., main effect of group; †† statistically different to placebo trial, i.e., main effect of supplement; ‡ statistically different to the placebo trial at the corresponding timepoint, for both WomenNC and WomenOC; ‡‡ statistically different to the placebo trial for WomenNC

GPx was lower in WomenOC (*β* [95% CI] = − 22.62 mU·mL^−1^ [− 41.32, − 3.91]; Fig. 3A). Plasma thiol concentrations were higher at all timepoints in the NAC trial compared to the placebo trial after supplement ingestion (i.e., after baseline), irrespective of group (all *p* < 0.001; *d* = 1.45 to 2.34; Fig. 3B). There was a *Time* (*β* = 3.43 mmol·L^−1^, [1.38, 5.49]) and a *Time* by *Supplement* effect on TAC (*β* = − 3.09 mmol·L^−1^ [− 6.04, − 0.14]). However, it was not clear whether TAC was statistically higher after the 1-km cycling TT (i.e., timepoint ‘post’) in the NAC trial compared to the placebo trial (*p* = 0.089; mean difference (MD) [95% CI] = 0.45 mmol·L^−1^ [− 0.07, 0.97]; *d* = 0.36; Fig. 3C).

There were effects of *Time* (*β* = 1.71 µmol·L^−1^ [0.24, 3.16]), *Supplement* (*β* = − 0.29 µmol·L^−1^ [− 0.56, − 0.02]) and *Time* by *Supplement* (*β* = -4.19 µmol·L^−1^ [− 6.24, − 2.09]) on total glutathione. Irrespective of group, total glutathione was statistically lower in the NAC trial compared to the placebo trial (Fig. 3D) at the end of the 40-min cycling task (*p* = 0.005; MD = − 0.47 µmol·L^−1^ [− 0.79, − 0.16]; *d* = − 0.40) and after the 1-km cycling TT (*p* < 0.001; MD = − 0.80 µmol·L^−1^ [− 1.20, − 0.41]; *d* = − 1.01).

There were *Time* (*β* [95% CI] = 13.47 mmol·L^−1^ [4.10, 22.84]) and *Group* (*β* = 12.00 mmol·L^−1^ [6.82, 17.17]) effects on MDA. In the placebo trial, MDA was statistically higher after the 1-km cycling TT compared to pre-exercise in WomenNC (*p* = 0.036; MD = 2.86 mmol·L^−1^ [0.14, 5.58]; *d* = 0.32) and WomenOC (*p* < 0.001; MD = 5.22 mmol·L^−1^ [1.98, 8.46]; *d* = 0.55). There was also a *Time* by *Group* by *Supplement* effect, with the ingestion of NAC attenuating the increase in MDA associated with exercise for WomenNC only (Fig. 3E). In the NAC trial, MDA was statistically lower after the 40-min cycling task (*p* = 0.018; MD [95% CI] = -3.28 mmol·L^−1^ [− 5.96, − 0.61]; *d* = − 0.73) and 1-km TT (*p* = 0.017; MD = − 3.69 mmol·L^−1^ [− 6.67, − 0.70]; *d* = − 0.50) compared to placebo trial in WomenNC. NAC ingestion did not attenuate the exercise-associated increase in MDA for WomenOC (Fig. 3E). There was an effect of *Time* on 3NT (*β*_time_^2^ = 9.25 mol·L^−1^ [2.78, 15.75]), but no effect on *Group* or *Supplement* (Fig. 3F).

CRP was higher in WomenOC (*β* [95% CI] = 1.53 mg·L^−1^ [0.76, 2.30]), but there was no effect of *Time* or *Supplement* (Fig. 3G). There was a *Time* effect on albumin (*β*_time_ = 53.53 g·L^−1^ [42.35, 64.72], but there was no effect of *Group* or *Supplement* (Fig. 3H).

### Time trial performance

**Fig. 4:**
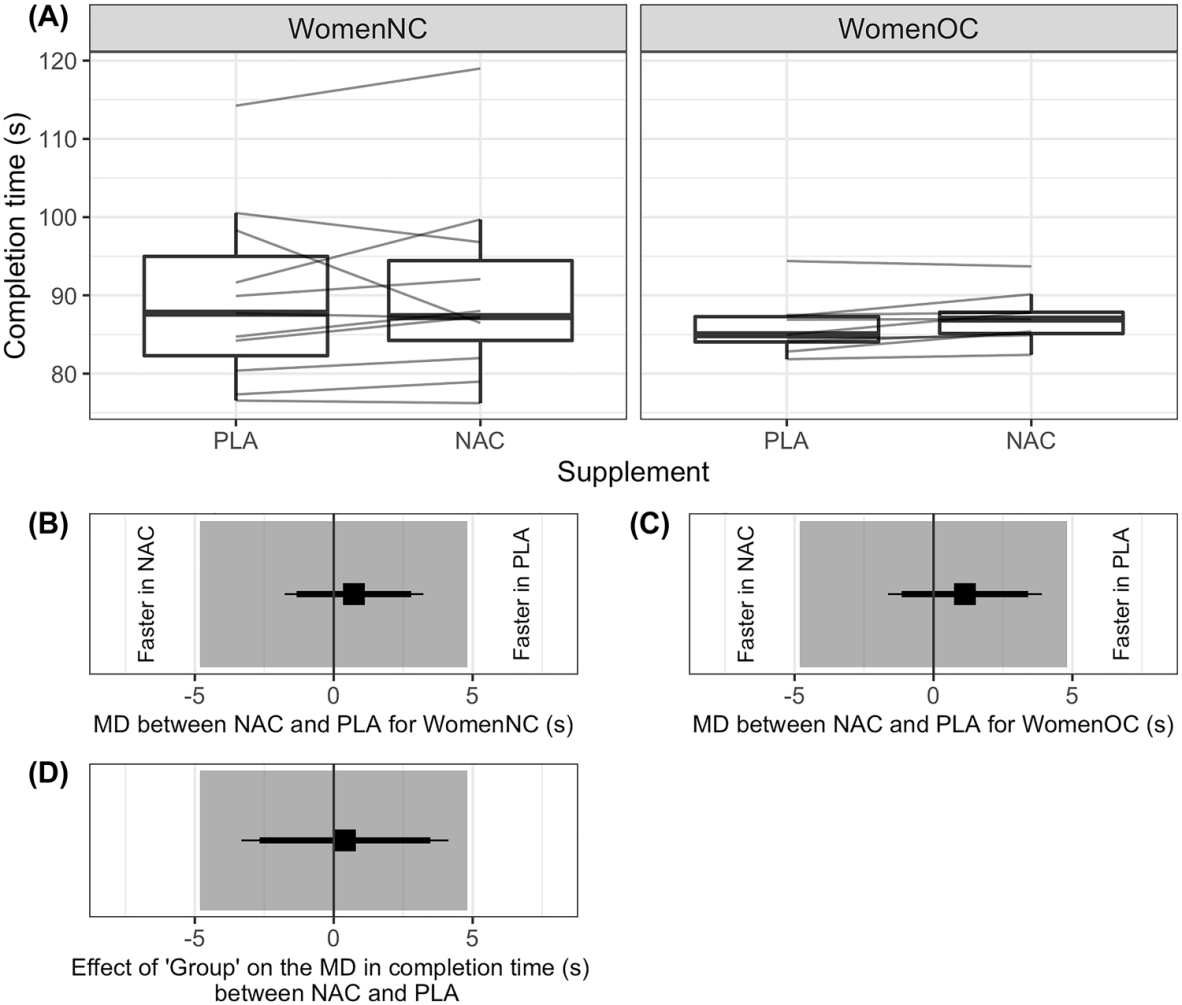
Completion time of a 1-km cycling time-trial by women naturally cycling (WomenNC) and women using combined, monophasic oral contraceptives (OC), after ingestion of a placebo beverage (PLA) and a beverage containing N-acetylcysteine (NAC; **A**). Mean difference (MD), as well as 90% (thick line) and 95% (thin line) confidence interval in completion time (s) between PLA and NAC in WomenNC (**B**) and WomenOC (**C**). The parameter estimate of ‘*Group*’ with 90% and 95% confidence interval, from a linear model used to fit the difference in time between PLA and NAC, for each participant in the study, where the difference (*y*_*i*_) was calculated using the equation: *y*_*i*_ = NAC_*i*_ – PLA_*i*_ (**D**). The grey shaded areas on Panels **B**, **C** and **D** show the region of statistical equivalence, determined from reliability data to be within the bounds of 0 ± 4.8 s. Dark grey vertical lines on Panels **B**, **C** and **D** indicate zero difference. Completion time was not statistically different, and was statistically equivalent, between the PLA and NAC trial for WomenNC and for WomenOC. There was no evidence of a *Group* effect on the difference in completion time between the PLA and NAC trial, with performance considered statistically equivalent between groups, as the 90% CI of the MD fell within the equivalence bounds of 0 ± 4.8 s

The 1-km TT completion times are presented in Fig. 4A. Completion time was statistically equivalent between the placebo and NAC trial for WomenNC (*d* = 0.05; Fig. 4B) and for WomenOC (*d* = 0.17; Fig. 4C). There was no *Group* effect on the difference in completion time between the NAC and placebo trial (*β* [95% CI] = 0.4 s [− 3.3, 4.1]), with completion time statistically equivalent between the groups (*β* [90% CI] = 0.4 s [− 2.7, 3.5]), based on the equivalence bounds of 0 ± 4.8 s (Fig. 4D). Participants adopted a negative-shaped pacing behaviour in all trials (supplementary material).

Mean [95% CI] mean power output during the placebo trial, 1-km TT for WomenNC and WomenOC was 276 W [238, 314] and 298 W [256, 340], respectively, whereas was 272 W [234, 310] and 285 W [243, 327], respectively, during the NAC trial. Peak heart rate for WomenNC and WomenOC during the placebo trial 1-km TT was 187 beat·min^−1^ [182, 192] and 185 beat·min^−1^ [179, 190], respectively, whereas was 186 beat·min^−1^ [181, 191] and 185 beat·min^−1^ [180, 191], respectively, during the NAC trial. Mean power output and peak heart rate were not different between groups in the placebo or NAC trial.

## Discussion

This study explored the effect of acute (1 h) oral NAC supplementation on the physiological and biochemical responses to exercise in WomenOC and WomenNC, in a double-blind, placebo-controlled, randomised, crossover experimental designed study. The results from this study indicate that (i) OC-use is associated with elevated oxidative stress, inflammation (CRP) and ventilation during cycling at a fixed, heavy-intensity compared to WomenNC, (ii) NAC attenuated the exercise-induced increase in lipid peroxidation (MDA) in WomenNC, but not for WomenOC, and (iii) NAC did not affect 1-km cycling TT performance in either group. These results provide experimental evidence of OC-use influencing selective physiological and biochemical responses to exercise. Elevated redox stress at rest and during exercise in WomenOC may indicate a rightwards shift on a redox hormesis curve (i.e., new physiological set point). The long-term implications of this on health, exercise adaptations and/or exercise recovery should be investigated.

## OC and exercise physiology (placebo trial)

Resting levels of MDA and CRP were significantly higher in WomenOC compared to WomenNC, and these between-group differences in MDA and CRP persisted during the fixed-intensity cycling bout, as well as after a 1-km cycling TT. The latter observations are novel as no previous research has compared exercise-induced oxidative damage or CRP between users and non-users of OC in a controlled exercise or performance task. Nonetheless, previous studies have reported similar findings in resting indices of lipid peroxidation and CRP (Cauci et al. 2016; Kowalska and Milnerowicz 2016; Larsen et al. 2020a; Larsen et al. 2018). This indicates that the high absolute concentration of MDA and CPR observed post-exercise in WomenOC are, in part, related to differences observed at rest. The higher presence of these blood biomarkers measured in women using OC at rest are likely to be derived from the metabolism of the exogenous sex steroids in the liver, which overwhelms conjugant pathways contributing to an increase in some liver proteins, RONS and oxidant damage (Chen et al. 1999).

In addition to OC-use, exercise can increase RONS and subsequent oxidant damage by interacting with cellular lipids (Merry and Ristow 2016). In support of this, the results suggest there was an exercise-induced increase in MDA in both groups in the placebo trial. The delta change was + 2.86 mmol·L^−1^ in WomenNC (relative increase: 51%), whereas was + 5.22 mmol·L^−1^ in WomenOC (relative increase 31%). Massart et al. (2012) did not find any between-group differences (OC users and non-users) in MDA concentrations after a combat (Judo) training session. However, the relative amount of work was not controlled for in the study design. The authors did note that the percent change (i.e., pre- to post-exercise) was significantly larger in non-users (pre: 0.04 ± 0.01 μg.mL^− 1^ to post: 0.07 ± 0.01 μg.mL^−1^) compared to OC users (pre: 0.09 ± 0.01 μg.mL^−1^ to post: 0.13 ± 0.01 μg.mL^−1^), but the absolute magnitude of change appears to be larger in the OC user group. Although, the presence of oxidative stress after exercise is suggested to widen the endpoints of physiological function and represent a better tolerance against subsequent stressors (Merry and Ristow 2016), it is unknown whether this is the case in women using OC without evidence of adaptation, and the available literature suggests chronically elevated oxidative stress is likely harmful for biological systems (Margaritelis et al. 2022). Therefore, the present results suggest that exercise may pose a further challenge for WomenOC to maintain redox homeostasis. Nonetheless, it remains unclear whether there is a physiological threshold or turn-point at which oxidant damage to macromolecules is classified as harmful/beneficial. Since accumulation of RONS during exercise can result in altered cellular redox signalling related to training adaptations and/or cellular function (Merry and Ristow 2016; Powers et al. 2016), and persistently high CRP and MDA production are associated with various pathological states (Baskol et al. 2006; Ridker et al. 2000), the long term implications of this altered redox homeostasis in WomenOC should be examined in greater detail. As such, it is suggested that mechanistic research is required to investigate the specific locations (tissues) or pathways (signalling molecules) for which OC-induced alterations in redox homeostasis at rest and during exercise have implications for training adaptations, recovery and possibly health in female athletes.

There was a similar exercise-induced increase in a blood biomarker that reflects protein nitration (i.e., 3NT) after 40-min fixed-intensity cycling and after 1-km cycling TT compared to baseline in both groups. 3NT can act as a surrogate marker for the formation of a strong oxidant; peroxynitrite (Shishehbor et al. 2003). This is due to the reaction of a superoxide anion with nitric oxide, which forms peroxynitrite. In the presence of peroxynitrite, the tyrosine residue of proteins can then undergo nitration, giving rise to 3NT. Previous research has reported plasma NO to be similar (Merki-Feld et al. 2002; Quinn et al. 2018), while tissue endothelial NO synthase mRNA levels to be higher (Cherney et al. 2007) in women using OC compared to women naturally cycling. Since the present study found no significant differences in 3NT between groups at rest or during exercise, this may indicate vascular oxidative stress related to superoxide and nitric oxide production and/or subsequent protein modification is not affected by OC use.

An increased tolerance to RONS production (e.g., upregulation of endogenous antioxidant defences) is an important aspect of physiological adaption. One enzymatic-antioxidant that is important for physiological function due to its ability to catalyse the breakdown of hydroperoxide is GPx. The present study found lower GPx at rest and during exercise in WomenOC compared to WomenNC. While GPx upregulates in response to skeletal muscle recruitment from regular exercise (Hellsten et al. 1996; Powers et al. 1994a), there was no exercise-induced change in GPx in response to exercise in either group. This could possibly be due to the duration of the exercise task (Powers et al. 1994b), or that the highest activity of GPx has been found in skeletal muscle fibres (Powers et al. 1994a), whereas we measured GPx in plasma. The homogeneity of the two groups’ characteristics (e.g., aerobic fitness) provides confidence that this finding of lower GPx in WomenOC is related to the use of exogenous sex steroids and/or suppression of endogenous sex steroids. Furthermore, previous research has noted similar findings of lower GPx in OC users at rest (Kowalska and Milnerowicz 2016). In contrast, there was no indication of between-group differences in non-enzymatic endogenous antioxidant defences including thiols, TAC, albumin, and total glutathione. However, previous literature has found lipid-soluble antioxidants, such as coenzyme Q_10_ (Palan et al. 2006), α-tocopherol (Palan et al. 2006) and β-carotene (De Groote et al. 2009; Palan et al. 2006) to be lower in women using OC compared to non-users. Collectively, our results, in conjunction with previous research suggest that only specific redox cycling pathways are affected by OC use and the impact of these specific pathways on biological function is relatively unexplored.

In addition to differences observed in the blood biomarkers, the ventilatory response to fixed-intensity cycling was different between groups (i.e., ventilation was 2.8 L·min^−1^ higher in WomenOC compared to WomenNC at Ex_40_), and the magnitude of difference was dependant on exercise duration. This finding has been noted during some studies (Assadpour et al. 2020; Quinn et al. 2018), but not all (Lei et al. 2019). Since there has been limited research to support OC-induced changes in ventilation, this study provides an important contribution to the literature. A change in ventilatory drive is suggested to occur from the stimulatory effect of (synthetic) progestin on mechanisms relating to respiration, such as increased sensitivity of chemoreceptors (Assadpour et al. 2020). This is also apparent for (natural) progesterone, whereby ventilation increases during the luteal phase of the menstrual cycle compared to the follicular phase (Dutton et al. 1989). Considering WomenNC were tested in the luteal phase when progesterone levels are at the highest concentration, it could suggest that progestin has more potent physiological effects related to respiration than progesterone. Future research could include measurements of pH and bicarbonate to interrogate any potential implications to acid–base balance in OC users. While ventilation was different between groups during fixed-intensity cycling, there were no differences observed in lactate, heart rate or perceived exertion between WomenNC and WomenOC during our exercise conditions, which is consistent with previous literature of exercise in similar temperature and humidity (Lei et al. 2019; Quinn et al. 2018).

## NAC and exercise physiology

Another key finding was that lipid peroxidation (MDA) significantly increased during the fixed-intensity cycling bout and 1-km TT in WomenOC after NAC supplementation. In contrast, NAC blunted the exercise-induced increase in MDA in WomenNC. Indeed, a blunting effect of NAC on MDA is supported by previous research in men, whereby antioxidant supplementation mitigates exercise-induced increases in markers of oxidative damage (Jówko et al. 2015; Medved et al. 2003; Slattery et al. 2014). Slattery et al. (2014) reported lower post-exercise F2-isoprostanes and thiobarbituric acid reactive substances in well-trained male triathletes after 9 days of NAC supplementation. In addition, Jówko et al. (2015) found lower exercise-induced MDA concentrations after 4 weeks of polyphenol supplementation in male college sprinters. In the present study, plasma thiols increased in both groups after 1 h of NAC ingestion, which remained elevated during exercise, and this provides evidence for higher antioxidant defence. Thus, the inability of NAC to blunt exercise-induced increases in MDA in WomenOC may reflect an OC-induced depletion of the body’s endogenous antioxidant capacity, likely resultant from excessive RONS production that is compounded from mechanisms related to both OC metabolism and exercise metabolism. It could also be postulated that a higher NAC dose in WomenOC may be required to observe a synonymous outcome. However, there is a greater risk of gastrointestinal discomfort with higher NAC doses (e.g., 140 mg·kg BM) (Ferreira et al. 2011). Plasma total glutathione was lower in both groups after the 40-min cycling task and 1-km TT during the NAC trial compared to placebo. Previous research in men has reported an increase in total glutathione after intravenous NAC supplementation (Medved et al. 2003) or that it remains stable after oral supplementation (Matuszczak et al. 2005), and this may be due to equivalent changes in the reduced to oxidised glutathione ratio (Ferreira et al. 2011; Matuszczak et al. 2005). Our finding of a reduction in total glutathione during exercise may represent sex differences during exercise or a translation of NAC into specific pro-oxidants that were quenched by glutathione (Devrim-Lanpir et al. 2021; Karimi et al. 2016). For example, thiols were elevated in the plasma from acute NAC ingestion, which may have resulted in mixed disulphide formation. Nonetheless, limited interpretation can be provided without other key redox biomarkers including reduced and oxidized glutathione.

## NAC and performance

Gross cycling efficiency declined in both groups across the fixed-intensity cycling bout. However, it was interesting to note that NAC may have lessened the slope of the decline in gross efficiency during the fixed-intensity cycling bout. While not clearly different from a statistical standpoint, with the 95% CI just including zero (i.e., 0.001), the difference in gross efficiency between NAC and Placebo after 40 min of cycling was 0.48% (i.e., 0.012% × 40 min), which could equate to meaningful performance improvement during a longer task, such as a 2-h performance test (Horowitz et al. 1994). Indeed, the bike dimensions, crank length, cadence and other environmental factors (e.g., temperature) that can alter the metabolic cost of cycling were controlled for within the study, thus supporting this to be a supplement effect. The mechanism that may support the preservation of cycling efficiency by NAC could be related to the maintenance of key ion transporter activity by reducing oxidant interference (McKenna et al. 2006; Medved et al. 2004a) and/or via improved regulation of calcium release within contracting myofibers (Andrade et al. 2001; Moopanar and Allen 2005).

Despite NAC acting to preserve gross efficiency during cycling, and possibly having an effect on performance during submaximal exercise, there appeared to be no effect of NAC supplementation on 1-km TT performance in WomenNC and WomenOC and thus, the data does not fully support our hypothesis. This null finding agrees with some studies conducted in men (Edwards et al. 2006; Medved et al. 2003), but not all, whereby several studies have found oral NAC supplementation to have ergogenic effects on performance (Cobley et al. 2011; Paschalis et al. 2018; Rhodes et al. 2019; Slattery et al. 2014), particularly in individuals with a glutathione deficiency (Paschalis et al. 2018). NAC has been reported to improve repeat sprint cycling performance (Slattery et al. 2014), performance of a repeated high-intensity intermittent shuttle (running) test (Cobley et al. 2011), maximum shuttle (running) sprint time (Rhodes et al. 2019) and cycling TT, Wingate and $\dot{\mathrm{V}}$ O_2_peak (Paschalis et al. 2018). Whereas, cycling TT performance (Edwards et al. 2006; Trewin et al. 2013), and high intensity cycling efforts (Medved et al. 2003) were not influenced by NAC supplementation. Since NAC is reported to be more effective in individuals with a glutathione deficiency, the incongruent findings may be related to baseline glutathione concentrations in the study participants (Paschalis et al. 2018). Other reasons for varying responses of NAC on performance may be related to a sex-specific difference in redox processes, the wide variety of exercise tasks including both sprint and endurance protocols, the length of supplementation period and/or the training status of recruited participants. While the fixed-intensity exercise bout performed before the 1-km TT allowed us to characterise the physiological responses to exercise between the two groups, submaximal pre-fatiguing protocols have also been amenable to a performance improvement by NAC (Cobley et al. 2011; Paschalis et al. 2018). Previous studies have employed intravenous NAC delivery or loading (multi-day) supplementation protocols, whereas the current study utilized a more practical acute dosing (single-day) regimen. While a significant increase in thiols and possible preservation in efficiency provided evidence for altered blood redox status after acute NAC supplementation, perhaps this did not translate to a discernible change in skeletal muscle intracellular redox state and function, as may occur with multi-day supplementation protocols.

A limitation of the study may be the selected blood biomarkers, as well as the sampling timepoints (Michailidis et al. 2007; Tsikas 2017). The TAC assay does not identify specific antioxidants present in plasma and the MDA assay used may potentially react with other aldehydes in plasma and not just those derived from lipid peroxides. However, including other assays and measuring multiple post-exercise samples (e.g., 30 min or 1 h into recovery) were not within the constraints of the study, and the crossover experimental design provided a means for addressing these considerations. The present study had rigorous inclusion criteria for WomenNC and employed a three-step method to determine the mid-luteal phase for the intervention trials. This selective recruitment strategy, as well as other contextual factors, such as pregnancy or changing OC formulas part-way through the study introduced high attrition rates, which limited participant numbers. Nonetheless, the nature of the study design (i.e., cross-over experimental) accounted for some of the individual variation. While the participants’ OC contained one of three different progestins, it is not anticipated that this would have influenced the between-group outcomes for our primary biological outcome measures (i.e., oxidative stress) (Cauci et al. 2020). However, other parameters such as ventilation may be influenced by the androgenicity or the concentration of the progestin and is a limitation to the current design. Finally, the results should be extrapolated to elite female athletes with caution as all participants were classified as recreationally trained.

### Conclusions

This study provides experimental evidence of OC-use influencing selective physiological and biochemical responses to exercise, specifically increased ventilation, MDA and CRP during heavy-intensity cycling compared to WomenNC. Another key finding was that acute NAC supplementation (i.e., 1 h prior to exercise) increased plasma thiols in both groups, which blunted the exercise-induced increase in MDA in WomenNC, but not in WomenOC. These findings provide preliminary evidence for increased strain on the endogenous antioxidant system during exercise when using OC, nonetheless, there was no deleterious effect on sprint cycling performance. Future investigations should explore the impact of OC-induced alterations to redox homeostasis on exercise adaptations, recovery from exercise and health in women.

## Supplementary Information

Below is the link to the electronic supplementary material.

Supplementary file1 (TIFF 12308 KB)

Supplementary file2 (PNG 114 KB)

## Abbreviations

3NT: 3-Nitrotyrosine
ABTS: 2,2'-Azino-bis(3-ethylbenzothiazoline-6-sulfonic acid)
BIC: Bayesian information criteria
BL: Baseline
CI: Confidence interval
CRP: C-reactive protein
CV: Coefficient of variation
Ex_20_: 20 Min into fixed-intensity exercise
Ex_40_: 40 Min into fixed-intensity exercise
GET: Gas exchange threshold
GPx: Glutathione peroxidase
LH: Luteinizing hormone
MD: Mean difference
MDA: Malondialdehyde
NAC: N-acetylcysteine
NULL: Experimental trial without any intervention
OC: Oral contraception
RPE: Rate of perceived exertion
RONS: Reactive oxygen and nitrogen species
SD: Standard deviation
TAC: Total antioxidant capacity
TT: Time trial
$\dot{\mathrm{V}}$O_2_peak: Peak oxygen uptake
WomenNC: Women with a natural, regular menstrual cycle
WomenOC: Women using combined, monophasic oral contraceptives
