# Supplementary figures and images for: Blood oxidative stress biomarkers in women: influence of oral contraception, exercise, and N-acetylcysteine
Source: Eur J Appl Physiol. 2022 Jun 8;122(8):1949–64. doi: 10.1007/s00421-022-04964-w (PMC9287208; doi:10.1007/s00421-022-04964-w)

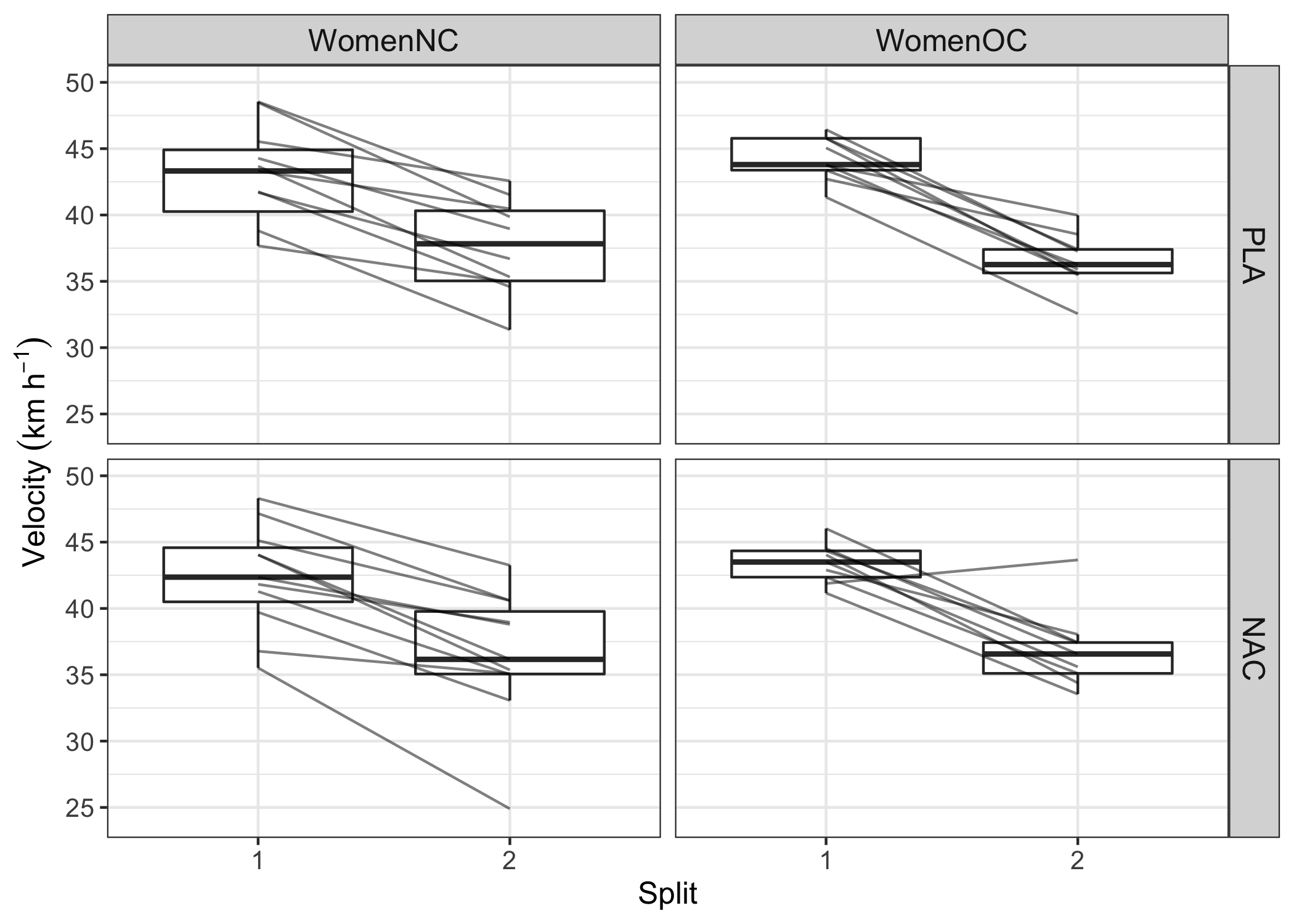

Supplement: Supplementary file 1 — Supplementary file1 (TIFF 12308 KB) [file 421_2022_4964_MOESM1_ESM.tiff]

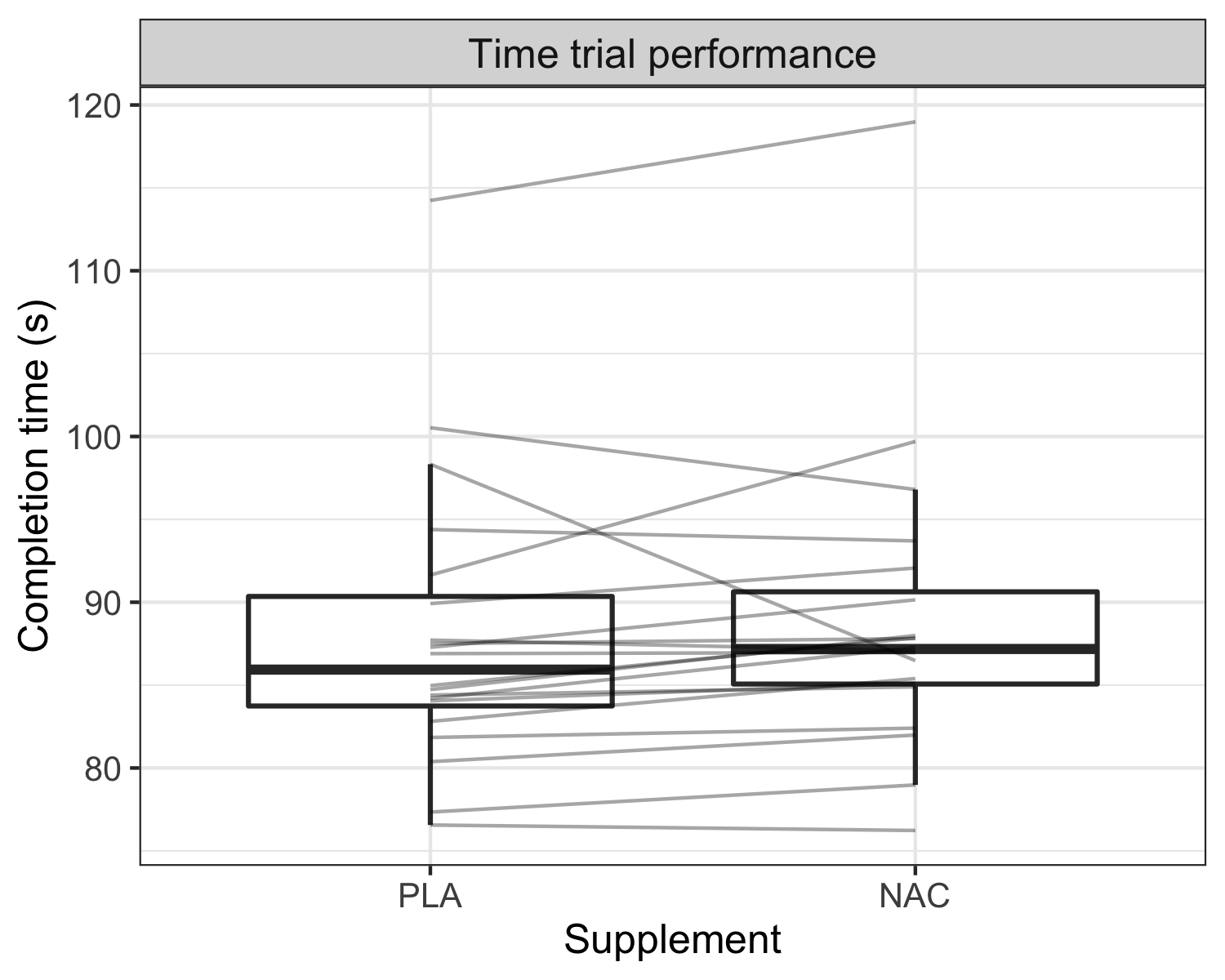

Supplement: Supplementary file 2 — Supplementary file2 (PNG 114 KB) [file 421_2022_4964_MOESM2_ESM.png]
